# Supplementary material for: Dynamic causal modelling highlights the importance of decreased self-inhibition of the sensorimotor cortex in motor fatigability
Source: Brain Struct Funct. 2024 Aug 28;229(9):2419–29. doi: 10.1007/s00429-024-02840-1 (PMC11611979; doi:10.1007/s00429-024-02840-1)
Supplement: Supplementary file 1 — Supplementary Material 1 [file 429_2024_2840_MOESM1_ESM.docx]

**Supplementary Material**

| **cluster-level** | | | | **peak-level** | | | | | **MNI-coord.** | **Anatomical Label** |  |
| --- | --- | --- | --- | --- | --- | --- | --- | --- | --- | --- | --- |
| p(FWE-corr) | p(FDR-corr) | equivk | p(unc) | p(FWE-corr) | p(FDR-corr) | T | equivZ | p(unc) | x,y,z {mm} |  |  |
|  |  |  |  |  |  |  |  |  |  |  |  |
| 0.000 | 0.000 | 2443 | 0.000 | 0.000 | 0.000 | 17.170 | 7.800 | 0.000 | 18–52 -22 | right Cb V/VI/VIIb |  |
|  |  |  |  | 0.000 | 0.004 | 9.490 | 6.060 | 0.000 | 6–74 -38 |  |  |
|  |  |  |  | 0.001 | 0.048 | 7.910 | 5.500 | 0.000 | 14–66 -40 |  |  |
| 0.000 | 0.000 | 2124 | 0.000 | 0.000 | 0.000 | 16.490 | 7.700 | 0.000 | -48 -22 50 | left BA1/BA2/BA3b/BA4a/BA4p |  |
|  |  |  |  | 0.000 | 0.000 | 14.250 | 7.280 | 0.000 | -42 -26 60 |  |  |
|  |  |  |  | 0.000 | 0.000 | 13.790 | 7.190 | 0.000 | -38 -20 52 |  |  |
| 0.000 | 0.000 | 498 | 0.000 | 0.000 | 0.002 | 9.990 | 6.220 | 0.000 | -52 -24 18 | left S2/OP1 |  |
| 0.000 | 0.000 | 620 | 0.000 | 0.000 | 0.004 | 9.660 | 6.120 | 0.000 | -2 -4 54 | left/right BA6 (SMA) |  |
|  |  |  |  | 0.010 | 0.297 | 6.740 | 5.000 | 0.000 | 6 -6 70 |  |  |
| 0.000 | 0.000 | 263 | 0.000 | 0.000 | 0.004 | 9.480 | 6.060 | 0.000 | -22 -2 -6 | left Pallidum/Putamen |  |
|  |  |  |  | 0.020 | 0.528 | 6.370 | 4.830 | 0.000 | -32 -6 0 |  |  |
| 0.000 | 0.000 | 425 | 0.000 | 0.000 | 0.007 | 9.090 | 5.930 | 0.000 | -46 6 -2 | left Central opercular cortex |  |
|  |  |  |  | 0.000 | 0.025 | 8.340 | 5.660 | 0.000 | -40 -2 16 |  |  |
|  |  |  |  | 0.025 | 0.624 | 6.250 | 4.770 | 0.000 | -58 6 2 |  |  |
| 0.002 | 0.070 | 32 | 0.035 | 0.001 | 0.031 | 8.170 | 5.600 | 0.000 | -58 4 28 | left BA6/BA44 |  |
| 0.000 | 0.006 | 80 | 0.002 | 0.004 | 0.176 | 7.150 | 5.190 | 0.000 | -26 -60 -22 | left CB V/VI |  |
| 0.002 | 0.070 | 32 | 0.035 | 0.006 | 0.221 | 6.970 | 5.110 | 0.000 | -14 -20 8 | left Thalamus |  |
| 0.002 | 0.070 | 34 | 0.030 | 0.010 | 0.297 | 6.730 | 5.000 | 0.000 | 40 − 12 56 | right BA6 |  |

*Supplementary Material 1: Peak activations during tapping. Areas highlighted in grey were associated with motor slowing and used as regions of interest in the DCM analysis (Bächinger et al. 2019).*


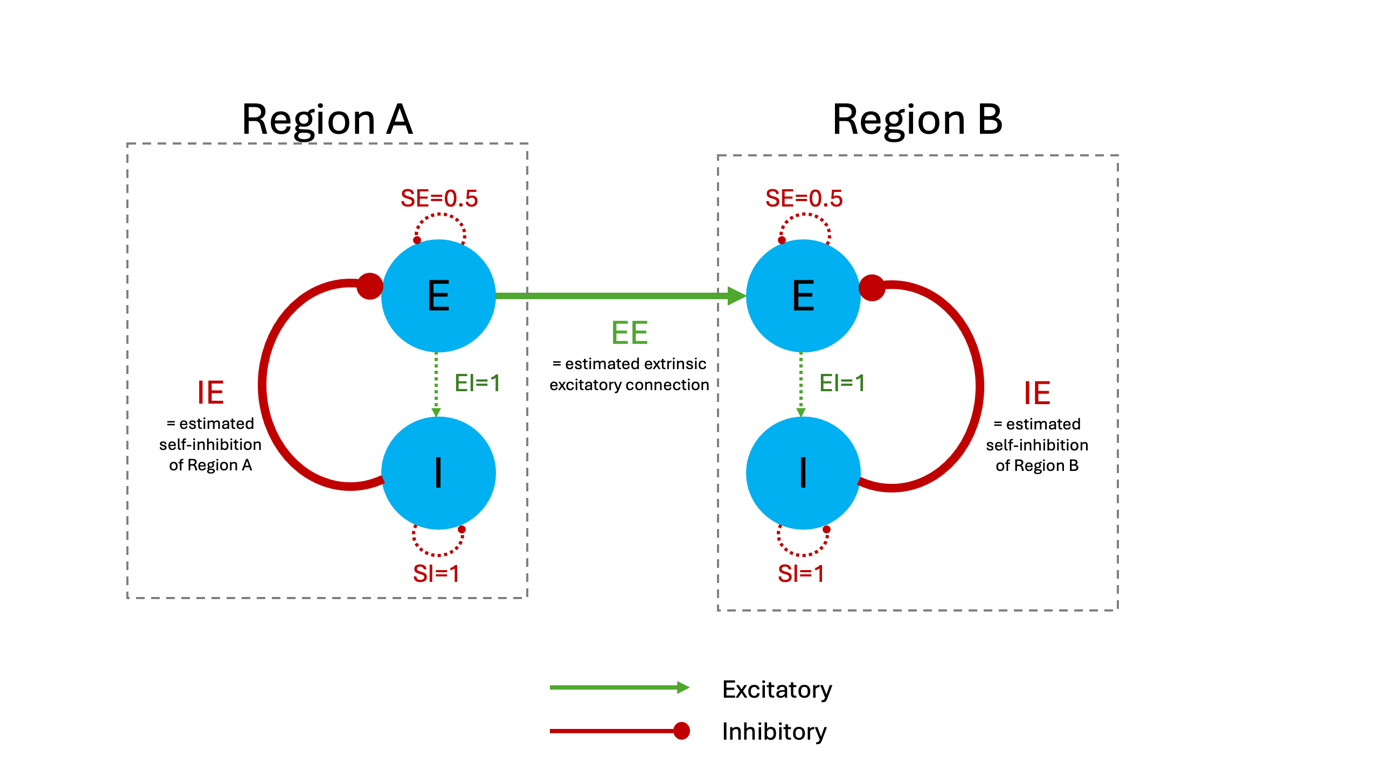


*Supplementary Material 2: Full DCM model of two regions (A and B) each with an excitatory (E) and an inhibitory (I) pool of neurons with excitatory (green) and inhibitory (red) connections. Note that DCM estimates only the connections from I to E (IE, solid red line), which represents the self-inhibition of a given region, and the facilitatory connection linking the excitatory pool of one region to the excitatory pool of another region (EE, solid green line). Other intrinsic connections are represented by pre-defined constants as indicated in the figure (see connections with dotted lines, EI: intrinsic excitatory to inhibitory; SI: intrinsic self-inhibition (inhibitory); SE: intrinsic self-inhibition (excitatory)). Adapted from Marreiros et al. 2008 and https://en.wikibooks.org/wiki/SPM/Two_State_DCM*

| Model-Nr. | SM1 Self-Mod. | SM1 -> PMd | SM1 -> SMA | PMd -> SM1 | PMd Self-Mod. | PMd -> SMA | SMA -> SM1 | SMA -> PMd | SMA Self-Mod. | DI to PMd | DI to SMA |
| --- | --- | --- | --- | --- | --- | --- | --- | --- | --- | --- | --- |
| Model001 | 0 | 0 | 0 | 1 | 0 | 0 | 0 | 0 | 0 | 0 | 1 |
| Model002 | 0 | 0 | 0 | 1 | 0 | 1 | 0 | 1 | 0 | 0 | 1 |
| Model003 | 0 | 0 | 0 | 1 | 0 | 0 | 0 | 0 | 0 | 1 | 0 |
| Model004 | 0 | 0 | 0 | 1 | 0 | 1 | 0 | 1 | 0 | 1 | 0 |
| Model005 | 0 | 0 | 0 | 1 | 0 | 0 | 0 | 0 | 0 | 1 | 1 |
| Model006 | 0 | 0 | 0 | 1 | 0 | 1 | 0 | 1 | 0 | 1 | 1 |
| Model007 | 0 | 0 | 0 | 0 | 0 | 0 | 1 | 0 | 0 | 0 | 1 |
| Model008 | 0 | 0 | 0 | 0 | 0 | 1 | 1 | 1 | 0 | 0 | 1 |
| Model009 | 0 | 0 | 0 | 0 | 0 | 0 | 1 | 0 | 0 | 1 | 0 |
| Model010 | 0 | 0 | 0 | 0 | 0 | 1 | 1 | 1 | 0 | 1 | 0 |
| Model011 | 0 | 0 | 0 | 0 | 0 | 0 | 1 | 0 | 0 | 1 | 1 |
| Model012 | 0 | 0 | 0 | 0 | 0 | 1 | 1 | 1 | 0 | 1 | 1 |
| Model013 | 0 | 0 | 0 | 1 | 0 | 0 | 1 | 0 | 0 | 0 | 1 |
| Model014 | 0 | 0 | 0 | 1 | 0 | 1 | 1 | 1 | 0 | 0 | 1 |
| Model015 | 0 | 0 | 0 | 1 | 0 | 0 | 1 | 0 | 0 | 1 | 0 |
| Model016 | 0 | 0 | 0 | 1 | 0 | 1 | 1 | 1 | 0 | 1 | 0 |
| Model017 | 0 | 0 | 0 | 1 | 0 | 0 | 1 | 0 | 0 | 1 | 1 |
| Model018 | 0 | 0 | 0 | 1 | 0 | 1 | 1 | 1 | 0 | 1 | 1 |
| Model019 | 0 | 0 | 0 | 1 | 1 | 0 | 0 | 0 | 0 | 0 | 1 |
| Model020 | 0 | 0 | 0 | 1 | 1 | 1 | 0 | 1 | 0 | 0 | 1 |
| Model021 | 0 | 0 | 0 | 1 | 1 | 0 | 0 | 0 | 0 | 1 | 0 |
| Model022 | 0 | 0 | 0 | 1 | 1 | 1 | 0 | 1 | 0 | 1 | 0 |
| Model023 | 0 | 0 | 0 | 1 | 1 | 0 | 0 | 0 | 0 | 1 | 1 |
| Model024 | 0 | 0 | 0 | 1 | 1 | 1 | 0 | 1 | 0 | 1 | 1 |
| Model025 | 0 | 0 | 0 | 0 | 0 | 0 | 1 | 0 | 1 | 0 | 1 |
| Model026 | 0 | 0 | 0 | 0 | 0 | 1 | 1 | 1 | 1 | 0 | 1 |
| Model027 | 0 | 0 | 0 | 0 | 0 | 0 | 1 | 0 | 1 | 1 | 0 |
| Model028 | 0 | 0 | 0 | 0 | 0 | 1 | 1 | 1 | 1 | 1 | 0 |
| Model029 | 0 | 0 | 0 | 0 | 0 | 0 | 1 | 0 | 1 | 1 | 1 |
| Model030 | 0 | 0 | 0 | 0 | 0 | 1 | 1 | 1 | 1 | 1 | 1 |
| Model031 | 0 | 0 | 0 | 1 | 1 | 0 | 1 | 0 | 1 | 0 | 1 |
| Model032 | 0 | 0 | 0 | 1 | 1 | 1 | 1 | 1 | 1 | 0 | 1 |
| Model033 | 0 | 0 | 0 | 1 | 1 | 0 | 1 | 0 | 1 | 1 | 0 |
| Model034 | 0 | 0 | 0 | 1 | 1 | 1 | 1 | 1 | 1 | 1 | 0 |
| Model035 | 0 | 0 | 0 | 1 | 1 | 0 | 1 | 0 | 1 | 1 | 1 |
| Model036 | 0 | 0 | 0 | 1 | 1 | 1 | 1 | 1 | 1 | 1 | 1 |
| Model037 | 1 | 0 | 0 | 1 | 0 | 0 | 0 | 0 | 0 | 0 | 1 |
| Model038 | 1 | 0 | 0 | 1 | 0 | 1 | 0 | 1 | 0 | 0 | 1 |
| Model039 | 1 | 0 | 0 | 1 | 0 | 0 | 0 | 0 | 0 | 1 | 0 |
| Model040 | 1 | 0 | 0 | 1 | 0 | 1 | 0 | 1 | 0 | 1 | 0 |
| Model041 | 1 | 0 | 0 | 1 | 0 | 0 | 0 | 0 | 0 | 1 | 1 |
| Model042 | 1 | 0 | 0 | 1 | 0 | 1 | 0 | 1 | 0 | 1 | 1 |
| Model043 | 1 | 0 | 0 | 0 | 0 | 0 | 1 | 0 | 0 | 0 | 1 |
| Model044 | 1 | 0 | 0 | 0 | 0 | 1 | 1 | 1 | 0 | 0 | 1 |
| Model045 | 1 | 0 | 0 | 0 | 0 | 0 | 1 | 0 | 0 | 1 | 0 |
| Model046 | 1 | 0 | 0 | 0 | 0 | 1 | 1 | 1 | 0 | 1 | 0 |
| Model047 | 1 | 0 | 0 | 0 | 0 | 0 | 1 | 0 | 0 | 1 | 1 |
| Model048 | 1 | 0 | 0 | 0 | 0 | 1 | 1 | 1 | 0 | 1 | 1 |
| Model049 | 1 | 0 | 0 | 1 | 0 | 0 | 1 | 0 | 0 | 0 | 1 |
| Model050 | 1 | 0 | 0 | 1 | 0 | 1 | 1 | 1 | 0 | 0 | 1 |
| Model051 | 1 | 0 | 0 | 1 | 0 | 0 | 1 | 0 | 0 | 1 | 0 |
| Model052 | 1 | 0 | 0 | 1 | 0 | 1 | 1 | 1 | 0 | 1 | 0 |
| Model053 | 1 | 0 | 0 | 1 | 0 | 0 | 1 | 0 | 0 | 1 | 1 |
| Model054 | 1 | 0 | 0 | 1 | 0 | 1 | 1 | 1 | 0 | 1 | 1 |
| Model055 | 1 | 0 | 0 | 1 | 1 | 0 | 0 | 0 | 0 | 0 | 1 |
| Model056 | 1 | 0 | 0 | 1 | 1 | 1 | 0 | 1 | 0 | 0 | 1 |
| Model057 | 1 | 0 | 0 | 1 | 1 | 0 | 0 | 0 | 0 | 1 | 0 |
| Model058 | 1 | 0 | 0 | 1 | 1 | 1 | 0 | 1 | 0 | 1 | 0 |
| Model059 | 1 | 0 | 0 | 1 | 1 | 0 | 0 | 0 | 0 | 1 | 1 |
| Model060 | 1 | 0 | 0 | 1 | 1 | 1 | 0 | 1 | 0 | 1 | 1 |
| Model061 | 1 | 0 | 0 | 0 | 0 | 0 | 1 | 0 | 1 | 0 | 1 |
| Model062 | 1 | 0 | 0 | 0 | 0 | 1 | 1 | 1 | 1 | 0 | 1 |
| Model063 | 1 | 0 | 0 | 0 | 0 | 0 | 1 | 0 | 1 | 1 | 0 |
| Model064 | 1 | 0 | 0 | 0 | 0 | 1 | 1 | 1 | 1 | 1 | 0 |
| Model065 | 1 | 0 | 0 | 0 | 0 | 0 | 1 | 0 | 1 | 1 | 1 |
| Model066 | 1 | 0 | 0 | 0 | 0 | 1 | 1 | 1 | 1 | 1 | 1 |
| Model067 | 1 | 0 | 0 | 1 | 1 | 0 | 1 | 0 | 1 | 0 | 1 |
| Model068 | 1 | 0 | 0 | 1 | 1 | 1 | 1 | 1 | 1 | 0 | 1 |
| Model069 | 1 | 0 | 0 | 1 | 1 | 0 | 1 | 0 | 1 | 1 | 0 |
| Model070 | 1 | 0 | 0 | 1 | 1 | 1 | 1 | 1 | 1 | 1 | 0 |
| Model071 | 1 | 0 | 0 | 1 | 1 | 0 | 1 | 0 | 1 | 1 | 1 |
| Model072 | 1 | 0 | 0 | 1 | 1 | 1 | 1 | 1 | 1 | 1 | 1 |
| Model073 | 0 | 1 | 0 | 0 | 0 | 0 | 0 | 0 | 0 | 0 | 1 |
| Model074 | 0 | 1 | 0 | 0 | 0 | 1 | 0 | 1 | 0 | 0 | 1 |
| Model075 | 0 | 1 | 0 | 0 | 0 | 0 | 0 | 0 | 0 | 1 | 0 |
| Model076 | 0 | 1 | 0 | 0 | 0 | 1 | 0 | 1 | 0 | 1 | 0 |
| Model077 | 0 | 1 | 0 | 0 | 0 | 0 | 0 | 0 | 0 | 1 | 1 |
| Model078 | 0 | 1 | 0 | 0 | 0 | 1 | 0 | 1 | 0 | 1 | 1 |
| Model079 | 0 | 0 | 1 | 0 | 0 | 0 | 0 | 0 | 0 | 0 | 1 |
| Model080 | 0 | 0 | 1 | 0 | 0 | 1 | 0 | 1 | 0 | 0 | 1 |
| Model081 | 0 | 0 | 1 | 0 | 0 | 0 | 0 | 0 | 0 | 1 | 0 |
| Model082 | 0 | 0 | 1 | 0 | 0 | 1 | 0 | 1 | 0 | 1 | 0 |
| Model083 | 0 | 0 | 1 | 0 | 0 | 0 | 0 | 0 | 0 | 1 | 1 |
| Model084 | 0 | 0 | 1 | 0 | 0 | 1 | 0 | 1 | 0 | 1 | 1 |
| Model085 | 0 | 1 | 1 | 0 | 0 | 0 | 0 | 0 | 0 | 0 | 1 |
| Model086 | 0 | 1 | 1 | 0 | 0 | 1 | 0 | 1 | 0 | 0 | 1 |
| Model087 | 0 | 1 | 1 | 0 | 0 | 0 | 0 | 0 | 0 | 1 | 0 |
| Model088 | 0 | 1 | 1 | 0 | 0 | 1 | 0 | 1 | 0 | 1 | 0 |
| Model089 | 0 | 1 | 1 | 0 | 0 | 0 | 0 | 0 | 0 | 1 | 1 |
| Model090 | 0 | 1 | 1 | 0 | 0 | 1 | 0 | 1 | 0 | 1 | 1 |
| Model091 | 0 | 1 | 0 | 0 | 1 | 0 | 0 | 0 | 0 | 0 | 1 |
| Model092 | 0 | 1 | 0 | 0 | 1 | 1 | 0 | 1 | 0 | 0 | 1 |
| Model093 | 0 | 1 | 0 | 0 | 1 | 0 | 0 | 0 | 0 | 1 | 0 |
| Model094 | 0 | 1 | 0 | 0 | 1 | 1 | 0 | 1 | 0 | 1 | 0 |
| Model095 | 0 | 1 | 0 | 0 | 1 | 0 | 0 | 0 | 0 | 1 | 1 |
| Model096 | 0 | 1 | 0 | 0 | 1 | 1 | 0 | 1 | 0 | 1 | 1 |
| Model097 | 0 | 0 | 1 | 0 | 0 | 0 | 0 | 0 | 1 | 0 | 1 |
| Model098 | 0 | 0 | 1 | 0 | 0 | 1 | 0 | 1 | 1 | 0 | 1 |
| Model099 | 0 | 0 | 1 | 0 | 0 | 0 | 0 | 0 | 1 | 1 | 0 |
| Model100 | 0 | 0 | 1 | 0 | 0 | 1 | 0 | 1 | 1 | 1 | 0 |
| Model101 | 0 | 0 | 1 | 0 | 0 | 0 | 0 | 0 | 1 | 1 | 1 |
| Model102 | 0 | 0 | 1 | 0 | 0 | 1 | 0 | 1 | 1 | 1 | 1 |
| Model103 | 0 | 1 | 1 | 0 | 1 | 0 | 0 | 0 | 1 | 0 | 1 |
| Model104 | 0 | 1 | 1 | 0 | 1 | 1 | 0 | 1 | 1 | 0 | 1 |
| Model105 | 0 | 1 | 1 | 0 | 1 | 0 | 0 | 0 | 1 | 1 | 0 |
| Model106 | 0 | 1 | 1 | 0 | 1 | 1 | 0 | 1 | 1 | 1 | 0 |
| Model107 | 0 | 1 | 1 | 0 | 1 | 0 | 0 | 0 | 1 | 1 | 1 |
| Model108 | 0 | 1 | 1 | 0 | 1 | 1 | 0 | 1 | 1 | 1 | 1 |
| Model109 | 1 | 1 | 0 | 0 | 0 | 0 | 0 | 0 | 0 | 0 | 1 |
| Model110 | 1 | 1 | 0 | 0 | 0 | 1 | 0 | 1 | 0 | 0 | 1 |
| Model111 | 1 | 1 | 0 | 0 | 0 | 0 | 0 | 0 | 0 | 1 | 0 |
| Model112 | 1 | 1 | 0 | 0 | 0 | 1 | 0 | 1 | 0 | 1 | 0 |
| Model113 | 1 | 1 | 0 | 0 | 0 | 0 | 0 | 0 | 0 | 1 | 1 |
| Model114 | 1 | 1 | 0 | 0 | 0 | 1 | 0 | 1 | 0 | 1 | 1 |
| Model115 | 1 | 0 | 1 | 0 | 0 | 0 | 0 | 0 | 0 | 0 | 1 |
| Model116 | 1 | 0 | 1 | 0 | 0 | 1 | 0 | 1 | 0 | 0 | 1 |
| Model117 | 1 | 0 | 1 | 0 | 0 | 0 | 0 | 0 | 0 | 1 | 0 |
| Model118 | 1 | 0 | 1 | 0 | 0 | 1 | 0 | 1 | 0 | 1 | 0 |
| Model119 | 1 | 0 | 1 | 0 | 0 | 0 | 0 | 0 | 0 | 1 | 1 |
| Model120 | 1 | 0 | 1 | 0 | 0 | 1 | 0 | 1 | 0 | 1 | 1 |
| Model121 | 1 | 1 | 1 | 0 | 0 | 0 | 0 | 0 | 0 | 0 | 1 |
| Model122 | 1 | 1 | 1 | 0 | 0 | 1 | 0 | 1 | 0 | 0 | 1 |
| Model123 | 1 | 1 | 1 | 0 | 0 | 0 | 0 | 0 | 0 | 1 | 0 |
| Model124 | 1 | 1 | 1 | 0 | 0 | 1 | 0 | 1 | 0 | 1 | 0 |
| Model125 | 1 | 1 | 1 | 0 | 0 | 0 | 0 | 0 | 0 | 1 | 1 |
| Model126 | 1 | 1 | 1 | 0 | 0 | 1 | 0 | 1 | 0 | 1 | 1 |
| Model127 | 1 | 1 | 0 | 0 | 1 | 0 | 0 | 0 | 0 | 0 | 1 |
| Model128 | 1 | 1 | 0 | 0 | 1 | 1 | 0 | 1 | 0 | 0 | 1 |
| Model129 | 1 | 1 | 0 | 0 | 1 | 0 | 0 | 0 | 0 | 1 | 0 |
| Model130 | 1 | 1 | 0 | 0 | 1 | 1 | 0 | 1 | 0 | 1 | 0 |
| Model131 | 1 | 1 | 0 | 0 | 1 | 0 | 0 | 0 | 0 | 1 | 1 |
| Model132 | 1 | 1 | 0 | 0 | 1 | 1 | 0 | 1 | 0 | 1 | 1 |
| Model133 | 1 | 0 | 1 | 0 | 0 | 0 | 0 | 0 | 1 | 0 | 1 |
| Model134 | 1 | 0 | 1 | 0 | 0 | 1 | 0 | 1 | 1 | 0 | 1 |
| Model135 | 1 | 0 | 1 | 0 | 0 | 0 | 0 | 0 | 1 | 1 | 0 |
| Model136 | 1 | 0 | 1 | 0 | 0 | 1 | 0 | 1 | 1 | 1 | 0 |
| Model137 | 1 | 0 | 1 | 0 | 0 | 0 | 0 | 0 | 1 | 1 | 1 |
| Model138 | 1 | 0 | 1 | 0 | 0 | 1 | 0 | 1 | 1 | 1 | 1 |
| Model139 | 1 | 1 | 1 | 0 | 1 | 0 | 0 | 0 | 1 | 0 | 1 |
| Model140 | 1 | 1 | 1 | 0 | 1 | 1 | 0 | 1 | 1 | 0 | 1 |
| Model141 | 1 | 1 | 1 | 0 | 1 | 0 | 0 | 0 | 1 | 1 | 0 |
| Model142 | 1 | 1 | 1 | 0 | 1 | 1 | 0 | 1 | 1 | 1 | 0 |
| Model143 | 1 | 1 | 1 | 0 | 1 | 0 | 0 | 0 | 1 | 1 | 1 |
| Model144 | 1 | 1 | 1 | 0 | 1 | 1 | 0 | 1 | 1 | 1 | 1 |
| Model145 | 0 | 1 | 0 | 1 | 1 | 0 | 0 | 0 | 0 | 0 | 1 |
| Model146 | 0 | 1 | 0 | 1 | 1 | 1 | 0 | 1 | 0 | 0 | 1 |
| Model147 | 0 | 1 | 0 | 1 | 1 | 0 | 0 | 0 | 0 | 1 | 0 |
| Model148 | 0 | 1 | 0 | 1 | 1 | 1 | 0 | 1 | 0 | 1 | 0 |
| Model149 | 0 | 1 | 0 | 1 | 1 | 0 | 0 | 0 | 0 | 1 | 1 |
| Model150 | 0 | 1 | 0 | 1 | 1 | 1 | 0 | 1 | 0 | 1 | 1 |
| Model151 | 0 | 0 | 1 | 0 | 0 | 0 | 1 | 0 | 1 | 0 | 1 |
| Model152 | 0 | 0 | 1 | 0 | 0 | 1 | 1 | 1 | 1 | 0 | 1 |
| Model153 | 0 | 0 | 1 | 0 | 0 | 0 | 1 | 0 | 1 | 1 | 0 |
| Model154 | 0 | 0 | 1 | 0 | 0 | 1 | 1 | 1 | 1 | 1 | 0 |
| Model155 | 0 | 0 | 1 | 0 | 0 | 0 | 1 | 0 | 1 | 1 | 1 |
| Model156 | 0 | 0 | 1 | 0 | 0 | 1 | 1 | 1 | 1 | 1 | 1 |
| Model157 | 1 | 1 | 0 | 1 | 1 | 0 | 0 | 0 | 0 | 0 | 1 |
| Model158 | 1 | 1 | 0 | 1 | 1 | 1 | 0 | 1 | 0 | 0 | 1 |
| Model159 | 1 | 1 | 0 | 1 | 1 | 0 | 0 | 0 | 0 | 1 | 0 |
| Model160 | 1 | 1 | 0 | 1 | 1 | 1 | 0 | 1 | 0 | 1 | 0 |
| Model161 | 1 | 1 | 0 | 1 | 1 | 0 | 0 | 0 | 0 | 1 | 1 |
| Model162 | 1 | 1 | 0 | 1 | 1 | 1 | 0 | 1 | 0 | 1 | 1 |
| Model163 | 1 | 0 | 1 | 0 | 0 | 0 | 1 | 0 | 1 | 0 | 1 |
| Model164 | 1 | 0 | 1 | 0 | 0 | 1 | 1 | 1 | 1 | 0 | 1 |
| Model165 | 1 | 0 | 1 | 0 | 0 | 0 | 1 | 0 | 1 | 1 | 0 |
| Model166 | 1 | 0 | 1 | 0 | 0 | 1 | 1 | 1 | 1 | 1 | 0 |
| Model167 | 1 | 0 | 1 | 0 | 0 | 0 | 1 | 0 | 1 | 1 | 1 |
| Model168 | 1 | 0 | 1 | 0 | 0 | 1 | 1 | 1 | 1 | 1 | 1 |
| Model169 | 0 | 0 | 0 | 0 | 0 | 0 | 0 | 0 | 0 | 0 | 1 |
| Model170 | 0 | 0 | 0 | 0 | 0 | 1 | 0 | 1 | 0 | 0 | 1 |
| Model171 | 0 | 0 | 0 | 0 | 0 | 0 | 0 | 0 | 0 | 1 | 0 |
| Model172 | 0 | 0 | 0 | 0 | 0 | 1 | 0 | 1 | 0 | 1 | 0 |
| Model173 | 0 | 0 | 0 | 0 | 0 | 0 | 0 | 0 | 0 | 1 | 1 |
| Model174 | 0 | 0 | 0 | 0 | 0 | 1 | 0 | 1 | 0 | 1 | 1 |
| Model175 | 1 | 0 | 0 | 0 | 0 | 0 | 0 | 0 | 0 | 0 | 1 |
| Model176 | 1 | 0 | 0 | 0 | 0 | 1 | 0 | 1 | 0 | 0 | 1 |
| Model177 | 1 | 0 | 0 | 0 | 0 | 0 | 0 | 0 | 0 | 1 | 0 |
| Model178 | 1 | 0 | 0 | 0 | 0 | 1 | 0 | 1 | 0 | 1 | 0 |
| Model179 | 1 | 0 | 0 | 0 | 0 | 0 | 0 | 0 | 0 | 1 | 1 |
| Model180 | 1 | 0 | 0 | 0 | 0 | 1 | 0 | 1 | 0 | 1 | 1 |

*Supplementary Material 3: Model space. The first six columns indicate whether motor slowing had a modulatory effect on the respective connections. The last three columns reflect to which nodes (SMA, PMd, or both) the driving input (DI) of tapping was set. Corresponding model families are listed here: Models 1–36: top-down models without self-inhibition of SM1; Models 37–72: top-down models with self-inhibition of SM1; Models 73–108: bottom-up models without self-inhibition of SM1; Models 109–144: bottom-up models with self-inhibition of SM1; Models 145–156:* selective premotor *models without self-inhibition of SM1; Models 157–168:* selective *premotor models with self-inhibition of SM1; Models 196 − 174: null models without self-inhibition of SM1; Models 175–180: null models with self-inhibition of SM1.*

| **Participant** | **Variance Explained** |
| --- | --- |
| Participant 1 | 21.07 |
| Participant 2 | 31.48 |
| Participant 3 | 42.67 |
| Participant 4 | 40.46 |
| Participant 5 | 45.65 |
| Participant 6 | 46.48 |
| Participant 7 | 23.48 |
| Participant 8 | 20.80 |
| Participant 9 | 24.23 |
| Participant 10 | 53.58 |
| Participant 11 | 6.98 |
| Participant 12 | 25.13 |
| Participant 13 | 54.06 |
| Participant 14 | 32.35 |
| Participant 15 | 29.60 |
| Participant 16 | 32.49 |
| Participant 17 | 37.01 |
| Participant 18 | 32.91 |
| Participant 19 | 19.65 |
| Participant 20 | 21.85 |
| Participant 21 | 53.83 |
| Participant 22 | 12.50 |
| Participant 23 | 32.84 |
| Participant 24 | 24.50 |

*Supplementary Material 4: Average variances explained by models of winning model family per participant. The variances explained by the models of the winning model family were averaged per participant.*

| **Participant** | **Model 1** | **Model 2** | **Model 3** | **Model 4** | **Variance explained 1** | **Variance explained 2** | **Variance explained 3** | **Variance explained 4** |
| --- | --- | --- | --- | --- | --- | --- | --- | --- |
| Participant 1 | 50 | 68 |  |  | 27.23 | 28.27 |  |  |
| Participant 2 | 126 |  |  |  | 34.59 |  |  |  |
| Participant 3 | 174 |  |  |  | 43.74 |  |  |  |
| Participant 4 | 34 |  |  |  | 43.7 |  |  |  |
| Participant 5 | 70 | 72 |  |  | 46.01 | 46.25 |  |  |
| Participant 6 | 106 | 124 | 142 |  | 46.62 | 46.7 | 46.76 |  |
| Participant 7 | 34 | 69 | 70 | 106 | 24.44 | 24.57 | 24.79 | 24.59 |
| Participant 8 | 52 |  |  |  | 39.5 |  |  |  |
| Participant 9 | 158 |  |  |  | 39.44 |  |  |  |
| Participant 10 | 16 |  |  |  | 56.97 |  |  |  |
| Participant 11 | 16 |  |  |  | 23.5 |  |  |  |
| Participant 12 | 144 |  |  |  | 31.41 |  |  |  |
| Participant 13 | 164 |  |  |  | 57.78 |  |  |  |
| Participant 14 | 36 | 52 | 72 |  | 34.73 | 34.54 | 34.87 |  |
| Participant 15 | 166 |  |  |  | 40.2 |  |  |  |
| Participant 16 | 16 | 52 | 69 |  | 36.94 | 36.79 | 36.85 |  |
| Participant 17 | 52 | 60 | 160 |  | 43.71 | 43.18 | 41.38 |  |
| Participant 18 | 166 |  |  |  | 32.88 |  |  |  |
| Participant 19 | 126 |  |  |  | 28.91 |  |  |  |
| Participant 20 | 72 |  |  |  | 24.23 |  |  |  |
| Participant 21 | 60 | 72 |  |  | 54.78 | 54.76 |  |  |
| Participant 22 | 68 |  |  |  | 12.53 |  |  |  |
| Participant 23 | 16 | 52 | 70 |  | 33.83 | 33.8 | 34.03 |  |
| Participant 24 | 34 | 70 | 106 | 142 | 30.63 | 29.95 | 29.46 | 30.36 |

*Supplementary Material 5: Variances explained by models in the Occam's window per participant. The model number with the variance explained for this participant are listed. The number of models in the Occam’s window varied across participant.*

*
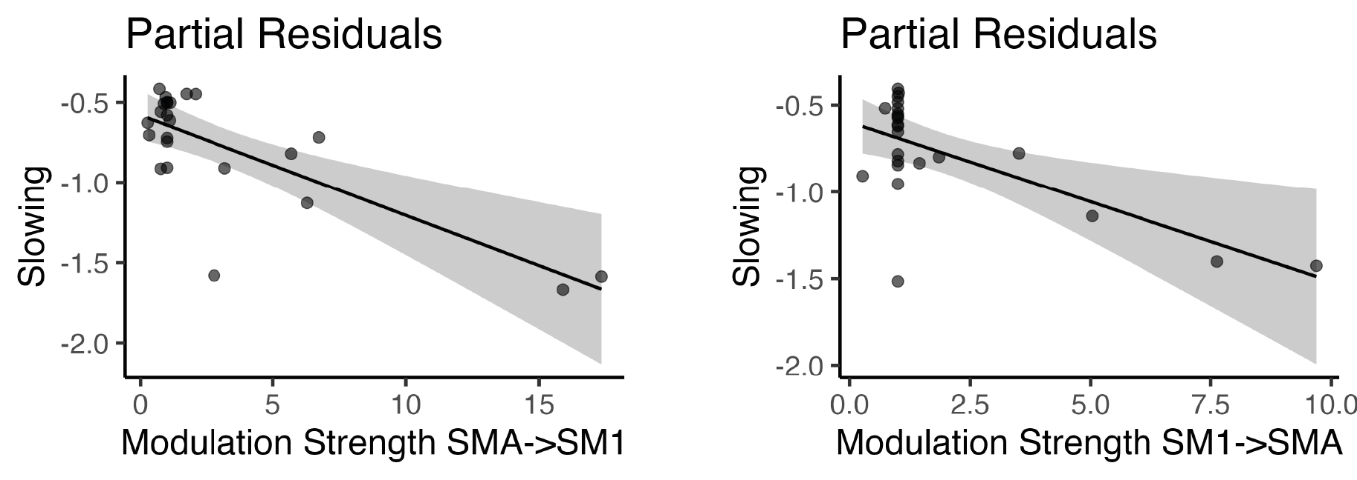
*

*Supplementary Material 6: Partial residuals of stepwise linear regression analysis. Both significant regressors seem to be driven by outliers.*
